# Supplementary material for: Health system strengthening in fragile and conflict-affected states: A review of systematic reviews
Source: PLoS One. 2024 Jun 14;19(6):e0305234. doi: 10.1371/journal.pone.0305234 (PMC11178226; doi:10.1371/journal.pone.0305234)
Supplement: S1 Annex — (DOCX) [file pone.0305234.s001.docx]

**S1 Annex: Search strategy**

1. **Scopus**

(TITLE-ABS-KEY((health W/2 (system? or care or service?)) OR (healthcare W/2 (system? or service?)))) AND (TITLE-ABS-KEY(("conflict affected") OR ("post conflict") OR ((fragil* or conflict* or weak or war or ("war torn")) W/3 (state? or countr* or territor* or region* or setting? or context? or situation?)) OR (humanitarian*))) AND ( LIMIT-TO ( DOCTYPE,"re" ) ) AND ( LIMIT-TO ( LANGUAGE,"English" ) )

1. **Web of science**
2. Search: ((((TS=(conflict-affected)) OR TS=(post-conflict)) OR TS=(postconflict) OR TS=((fragil* or conflict* or war or war-torn) NEAR/3 (state? or countr* or territor* or region? or setting? or context? or situation?))) OR TS=(humanitarian*))
3. Search: (((TS=((health NEAR/2 (system? or service? or care)))) OR TS=(healthcare NEAR/2 (system? or service?))))
4. # Searches: Search: #1 AND #2 and Review Article (Document Types) and English (Languages)
5. **Cochrane Library**

**ID Search**

#1 MeSH descriptor: [Health Care Economics and Organizations] explode all trees

#2 MeSH descriptor: [Health Care Facilities, Manpower, and Services] explode all trees

#3 MeSH descriptor: [Delivery of Health Care] explode all trees

#4 (health NEAR/2 (system? or care or service?)):ti,ab,kw OR (healthcare NEAR/2 (system? or service?)):ti,ab,kw

#5 ((health or healthcare or care) NEAR/2 (primary or essential)):ti,ab,kw

#6 {OR #1-#5}

#7 (conflict NEAR/2 affected):ti,ab,kw OR (post NEXT conflict):ti,ab,kw OR (postconflict):ti,ab,kw

#8 ((fragil* or conflict* or weak or war or (war NEXT torn)) NEAR/2 (state? or countr* or territor* or region* or setting? or context? or situation?)):ti,ab,kw

#9 (humanitarian*):ti,ab,kw OR (complex NEAR/2 (emergency or emergencies)):ti,ab,kw

#10 {OR #7-#9}

#11 #6 AND #10

1. **Ovid (Medline, Embase and Global Health)**
2. exp "health care (non mesh)"/
3. exp Patient Care/
4. (health adj3 (system? or care or service?)).ti,ab,kw.
5. (healthcare adj3 (system? or service?)).ti,ab,kw.
6. 1 or 2 or 3 or 4
7. 3 or 4
8. (conflict adj3 affected).ti,ab,kw.
9. post conflict.ti,ab,kw.
10. postconflict.ti,ab,kw.
11. ((fragil* or conflict* or weak or war or war NEXT torn) adj3 (state? or countr* or territor* or region* or setting? or context? or situation?)).ti,ab,kw.
12. humanitarian*.ti,ab,kw.
13. or/7-11
14. (((systematic or state-of-the-art or scoping or literature or umbrella) adj (review* or overview* or assessment*)) or "review* of reviews" or meta-analy* or metaanaly* or ((systematic or evidence) adj1 assess*) or "research evidence" or metasynthe* or meta-synthe*).tw. or exp Review Literature as Topic/ or exp Review/ or Meta-Analysis as Topic/ or Meta-Analysis/ or "systematic review"/
15. 5 and 12 and 13
16. 14 use medall
17. exp health care/
18. exp "health care facilities and services"/
19. exp health care system/
20. 16 or 17 or 18
21. 6 or 19
22. (((systematic or state-of-the-art or scoping or literature or umbrella) adj (review* or overview* or assessment*)) or "review* of reviews" or meta-analy* or metaanaly* or ((systematic or evidence) adj1 assess*) or "research evidence" or metasynthe* or meta-synthe*).tw. or systematic review/ or "systematic review (topic)"/ or meta analysis/ or "meta analysis (topic)"/
23. 12 and 20 and 21
24. 22 use oemezd
25. exp health care/
26. exp health services/
27. 6 or 24 or 25
28. 12 and 13 and 26
29. 27 use cagf
30. 15 or 23 or 28
31. remove duplicates from 29
32. limit 30 to english language
