# Supplementary material for: Health system strengthening in fragile and conflict-affected states: A review of systematic reviews
Source: PLoS One. 2024 Jun 14;19(6):e0305234. doi: 10.1371/journal.pone.0305234 (PMC11178226; doi:10.1371/journal.pone.0305234)
Supplement: S2 Annex — (DOCX) [file pone.0305234.s002.docx]

**S2 Annex: Summary of the main findings**

| **Author/s** | **Foundations** | **Processes of Care** | **Quality Impacts** |
| --- | --- | --- | --- |
| Jordan et al., 2021 | - Emphasis on foundation of care than the process and quality impacts - The role of cultural norms of patients in shaping healthcare services - The role of community involvement to improve access and quality of care - The role of people-centred governance approach - Adoption of performance-based financing - Predictors of poor-quality care | - Poor quality care as major barrier to health systems - The lack of continuity of care; and poor patient experience as a quality concern | - Impact of lack of trust in health system - The role of quality of care in patient satisfaction - User experience in terms of discrimination and poor communication - The role of financial support for better access and healthcare utilisation - Impact of political instability on utilisation of healthcare services - The economic burden of poor-quality care has on both patients and health systems - The role of free or subsidised healthcare services to reduce financial burden and facilitating financial risk protection |
| Durrance-Bagale et al., 2020 | - The need for coordinated, integrated, and cooperative responses to crises situations at all levels - The role of displaced people in promoting better health - The role of non-health humanitarian clusters to improving health outcomes - The role of cultural norms of patients in shaping healthcare services - The need of understanding local circumstances or context and involving community leaders and displaced people, and the use of networks of organisations for community-based activities | - The need and role of integration healthcare services | - The role of cash transfers in promoting health and quality of life - The role of integrating healthcare services - Contribution of non-health humanitarian clusters to improving health outcomes |
| Lassi et al., 2015 | - The role of cultural norms of patients in shaping healthcare services - The different healthcare provision platforms for health delivery mechanisms: community based, outreach and facility-based services, and their impacts - Strategies and importance of strengthening facility-based services |  |  |
| Asgary et al., 2022 | - The role of cultural norms of patients in shaping healthcare services - The role of training the community, particularly refugees to address gaps in accessing care, and promote better health - The use of different NCDs care models, and barriers to accessing them - Barriers to implementation/success of new intervention models - The need for strengthening medication and supplies - The initiatives to build the capacity of healthcare services | - NCDs prevention and treatment strategies | - Influence of acceptability of healthcare services - The role of integration of services within existing systems |
| Vivalya et al., 2022 | - Burden and predictors of mental disorders in crisis situations - The role of community involvement in mental healthcare provision and improved health outcome - The role of establishing a strong relationship between modern mental healthcare and traditional or religious healers - The need of understanding local circumstances when designing new interventions - The importance of new model of mental healthcare services - A model for improved mental health services | - The need, challenges and facilitators of integrating healthcare services - The need for adapting national guidelines into the local context | - The lack and poor provision of care affecting mental health outcomes - Strategies to improve access to mental healthcare services |
| Homer et al., 2022 | - The impact of conflict on healthcare workers and health facilities - Community involvement in improving access, quality and healthcare utilisation - The role of community to provide a safe environment for healthcare workers - The role of the community in financially supporting the health system - Barriers and challenges of health system governance and supervision - The impact of culture and gender norms on female healthcare workers recruitment and training - The need to coordinate with traditional healthcare providers - The need for workforce planning - The use of people-centred governance approach | - Impacts of dangerous roads to healthcare facilities on accessing care and referral | - The effect of community's lack of trust in healthcare workers - The role of trust as facilitator and obstacle to the quality of care |
| Lokot et al., 2022 | - The role of refugees and asylum-seekers in health promotion and provision of care - Barriers and facilitators of health system governance - Problems with transparency, accountability, lack of trust in government agencies; and unethical practices - The need for collaboration between different stakeholders and community engagement - Mechanisms and models of coordination between organisations, agencies, and bodies that finance healthcare services, including cluster approach - Structural reforms in the adoption of service delivery - The role of decentralisation and people-centred governance approach - Challenges of the lack and restrictive healthcare policies - The need and advantage of support from higher/central level - The role of donors in health system governance - The low-quality emergency on-the-job training by NGOs and other bodies to fill government’s gap |  |  |
| Casey, 2015 | - The role of refugees and asylum-seekers in health promotion and provision of care - The importance of training community health workers, especially women - The challenges of contextual constraints and feasibility opportunities in provision of adequate healthcare services - The use of mobile clinics and trained mobile healthcare workers - The higher rates of healthcare services interruption post-disaster - Association of female community health workers with increased skilled birth attendance - The challenging nature of collecting data in humanitarian settings |  | - The role of financial support for better access and healthcare utilisation - The possibilities of implementing healthcare services in challenging settings, and their utilisation - The role of free or subsidised healthcare services |
| Durrance-Bagale et al., 2022 | - The role of community support in improving access, utilisation, and provision of care - The role of local community groups and leaders in health promotion and facilitating new health system interventions/solutions - The role of improved governance; and people-centred health systems governance for accountability and improved health system functions - The risks of implementing ‘aid’ projects without engaging the community - The role of traditional community groups in mediating between different actors | - The use of materials in local languages for better communication |  |
| Miyake et al., 2017 | - Challenges and consequences of conflict on health professional training/education, healthcare workers deployment and retention - Challenges and facilitators of running health professional education programme and accreditation - Influence of external actors, and governments on health professional education and training - Training community health workers/task-shifting to address the shortage of health workforce - Selection process and criteria for community health workers trainees - The role and advantage of the community in nomination of candidates - Challenges of female community health workers and trainees - Community health workers lack of health-system support after deployment - The lack of strict monitoring and supervision system, particularly in health facilities in rural areas - Reduced number of capable managers and supervisors’, post-conflict |  |  |
| Ismail et al., 2022 | - The role of community engagement in improving access to healthcare services - The use of prior networks and infrastructure for better provision of healthcare services - The role of different health financing strategies in facilitating health system reforms and services - The impact of adopting pay-for-performance - The role of donor funding, governance, partnerships, stakeholders’ engagement, and coordination for improved healthcare service delivery - The use of multiple healthcare service delivery pathways - The need for preparedness before disease outbreak - The need for considering contextual factors while planning new interventions - Barriers in provision of care such as healthcare worker payments | - The need for integration of childhood immunisations - Strategies to develop positive user experience |  |
| Rayes et al., 2021 | - Challenges and facilitators of workforce, and return and reintegration of healthcare workers - Impact of returning healthcare workers on existing workers, and the need for comprehensive national health workforce plan - Contribution of community organisation in the return of migrated healthcare workers - Push and pull-factors like higher salaries, better incentives, and favourable working environment for healthcare workers in conflict-affected health systems |  |  |
| Roome et al., 2014 | - Consequence of conflict on number and distribution of workforce - Challenges of human resource management and employment policies - The effect of poor human resources data on payment reforms - Challenges and facilitators of recruitment and retention of healthcare workers - Challenges and facilitators of healthcare workers training standards and quality assurance - Consequence of compromised quality and standards in education and training on the quality of healthcare workers produced - Unintended consequences of NGOs, aid organisations and private institution on the workforce market - Recruitment of expatriates and their consequences - Impacts of limited supportive supervision and necessary infrastructure, equipment and supplies on health professional education - Task shifting and training community health workers to mitigate shortages of trained and qualified healthcare workers - Challenges of female community health workers - The compromised quality ad hoc trainings by NGOs, aid agencies, and local providers - Policies and interventions to strengthen management and supervision capacity |  |  |
| Bertone et al., 2018 | - The use of performance-based financing (PBF) - Factors determining adoption and development of PBF |  |  |
| Chol et al., 2018 | - The role of health systems decentralisation - The role of different health financing strategies in facilitating health system reforms and services - Innovation related to health workforce including task shifting and training community health workers - Reasons and issues with recruitment of expatriates - The role of partnerships, stakeholders’ engagement, and coordination - Governments, NGOs and other donors as barriers and facilitators - The role of decentralisation and people-centred health systems governance |  | - The role of reducing out-of-pocket expenditure and free service for the poor as part of health system reforms |
| Akl et al., 2015 | - Mechanisms and models of coordination in humanitarian crises - The role of cluster approach in coordinating improving access to healthcare services - Information coordination, particularly the use of ICT, to support health and medical care during disaster response |  |  |
| Lotfi et al., 2016 | - The mechanisms and different models of coordination between organisations, agencies, and bodies providing or funding healthcare - services (the cluster approach, the 4Ws, the Sphere Project, 5x5 model, a model of information coordination, and Coordination for funding healthcare services) - The role of creating a reliable and cost-effective financial monitoring system |  |  |
| van Daalen et al., 2022 | - The use/application of cash transfers by governments and humanitarian agencies/NGOs for different purposes |  | - The role of cash transfers for promoting better health outcomes, quality of life and well-being; and improved healthcare utilisation - Potential negative impacts of cash transfers implementation - The role of cash transfers in reducing stress of financial burden |
| Schmid et al., 2022 | - Barriers to access to care in displaced population - Provision of healthcare in temporary settings, particularly camps, in crises settings - The need/importance of understanding local circumstances and stage of the humanitarian crisis when designing model of care - Unregulated private sector, and impact of income levels on its utilisation | - The presence of non-standardised and insufficient integration of healthcare services - The role of more accessible and affordable primary healthcare service provisions in temporary settings - The lack of understanding of referral processes | - The role of patients’ perception and trust in the health system in influencing care seeking behaviour - The use of private sector facilities - The role of perceived quality of care, and availability of healthcare workers and medicines on patients’ choice of services |
| Werner et al., 2022 | - The key functions of community health workers in in post-conflict settings - The role of trained community health workers in improving access and provision of care |  |  |
| Ruby et al., 2015 | - The importance of capacity-building and preparation of healthcare workers and staff for effective healthcare services - The success of algorithm-based interventions - The need for including NCDs medications on essential medication lists - The use of monitoring people using electronic medical records - Clinical outcomes and effectiveness of specific NCDs management programmes - The use of traditional treatment for improved health outcome |  |  |
| Lin et al., 2022 | - Challenges and unique consequence of conflict on workforce - Factors affecting intention to migrate or return from migration - Reasons healthcare workers to remain in a post-conflict setting - Challenges and facilitators (pros and cons) of returning migrant healthcare workers - Challenges and facilitators of providing health professional education and accreditation - Task shifting and training community health workers to mitigate shortages of trained and qualified healthcare workers - Challenges of female community health workers and trainees, and their effectiveness - Key lessons and successes from existing approaches and interventions to retain healthcare workers - Deployment, flight (to private and NGO healthcare providers) and retention of healthcare workers - The situation of post-conflict healthcare worker labour market disequilibrium - The impact of limitations of supportive supervision and infrastructure, equipment and supplies on health professional education |  |  |
| Dobiesz et al., 2022 | - Barriers and interventions of maintaining health professional education during war/conflict - Consequence of conflict on number and distribution of workforce - Influence of external actors, and the government on education and training - The low-quality emergency on-the-job training by NGOs and other bodies to fill the training gap - The impact of limitations of supportive supervision and necessary infrastructure, equipment and supplies on health professional education |  |  |
| Beek et al., 2017 | - Challenges of conflict and war in providing and maintaining health professional training and education - Challenges and facilitators of transferring knowledge and skills gained from training to the workplace and its maintenance over time |  | - The impact of lack trust towards healthcare providers - The impact of lack of available medicines in public healthcare service providers - The need for building relationships with traditional community healthcare providers to improve communication links |
| Abujaber et al., 2022 | - The importance of supportive supervision of lay healthcare workers - The different type and capacities of supportive supervision - The impacts of limitations of supportive supervision and necessary infrastructure, equipment and supplies on healthcare services and health professional education |  |  |
| Bowsher et al., 2021 | - The use of eHealth for clinical management, healthcare, and information management - eHealth interventions for health professional education - Production of eHealth initiatives - The role of technological innovations in addressing health challenges |  |  |
| Winders et al., 2021 | - The use of mHealth in education, data acquisition and data quality - The use of mHealth in monitoring symptoms using patient care devices - The use of novel patient care devices - The impact of mHealth on epidemic surveillance - The use of remote telehealth support |  | - The role of cash transfers in improving food security, nutrition, and wellbeing |
