# Supplementary material for: Health system strengthening in fragile and conflict-affected states: A review of systematic reviews
Source: PLoS One. 2024 Jun 14;19(6):e0305234. doi: 10.1371/journal.pone.0305234 (PMC11178226; doi:10.1371/journal.pone.0305234)
Supplement: S4 Annex — (DOCX) [file pone.0305234.s004.docx]

**S4 Annex: Methodological quality appraisal using the Joanna Briggs Institution (JBI) Critical Appraisal Checklist for Systematic Reviews and Research Syntheses**

| **Study Id.** | **1. Is the review question clearly and explicitly stated?**  **25** | **2. Were the inclusion criteria appropriate for the review question?**  **25** | **3. Was the search strategy appropriate?**  **12** | **4. Were the sources and resources used to search for studies adequate?**  **20** | **5. Were the criteria for appraising studies appropriate?**  **12** | **6. Was critical appraisal conducted by two or more reviewers independently?**  **0** | **7. Were there methods to minimize errors in data extraction?**  **13** | **8. Were the methods used to combine studies appropriate?**  **26** | **9. Was the likelihood of publication bias assessed?** | **10. Were recommendations for policy and/or practice supported by the reported data?**  **22** | **11. Were the specific directives for new research appropriate?**  **25** | **Total ‘yes’ answer** | **Overall appraisal** |
| --- | --- | --- | --- | --- | --- | --- | --- | --- | --- | --- | --- | --- | --- |
| Jordan et al., 2021 | Yes | Yes | Yes | Yes | Yes | Unclear | Unclear | Yes | NA | Yes | Yes | 8 | High |
| Durrance-Bagale et al., 2020 | Yes | Yes | Yes | Yes | No | Unclear | Unclear | Yes | NA | Yes | Unclear | 6 | Moderate |
| Lassi et al., 2015 | Yes | Yes | Yes | Yes | No | Unclear | Yes | Yes | NA | Yes | Yes | 8 | High |
| Asgary et al., 2022 | Yes | Yes | Yes | Yes | Unclear | No | Unclear | Yes | NA | Yes | Yes | 7 | Moderate |
| Vivalya et al., 2022 | Unclear | Unclear | Unclear | No | No | Unclear | Unclear | Yes | NA | Yes | Yes | 3 | Low |
| Homer et al., 2022 | Yes | Yes | Unclear | Yes | Yes | Unclear | Yes | Yes | NA | Yes | Yes | 8 | High |
| Lokot et al., 2022 | Yes | Yes | Unclear | Yes | Yes | Unclear | Yes | Yes | NA | Unclear | Yes | 7 | Moderate |
| Casey, 2015 | Yes | Yes | Unclear | No | Yes | Unclear | Unclear | Unclear | NA | Yes | Unclear | 4 | Low |
| Durrance-Bagale et al., 2022 | Yes | Yes | Yes | No | No | No | Unclear | Yes | NA | Yes | Yes | 6 | Moderate |
| Miyake et al., 2016 | Yes | Yes | Yes | Yes | Yes | No | Yes | Yes | NA | Yes | Yes | 9 | High |
| Ismail et al., 2022 | Yes | Yes | Yes | Yes | Yes | Unclear | Yes | Yes | NA | Yes | Yes | 9 | High |
| Rayes et al., 2021 | Yes | Yes | No | Yes | No | No | Unclear | Yes | NA | Yes | Yes | 6 | Moderate |
| Roome et al., 2014 | No | Yes | Unclear | Yes | No | Unclear | Unclear | Yes | NA | Yes | Yes | 5 | Moderate |
| Bertone et al., 2018 | Yes | Yes | No | No | No | No | Unclear | Yes | NA | Yes | Yes | 5 | Moderate |
| Chol et al., 2018 | Yes | Yes | Unclear | Yes | No | No | Yes | Yes | NA | Unclear | Yes | 6 | Moderate |
| Akl et al., 2015 | Yes | Yes | Yes | Yes | Yes | Unclear | Yes | Yes | NA | Yes | Yes | 9 | High |
| Lotfi et al., 2016 | Yes | Yes | Yes | Yes | No | No | Yes | Yes | NA | Yes | Yes | 8 | High |
| Van Daalen et al., 2022 | Yes | Yes | Yes | Yes | Yes | Unclear | Yes | Yes | NA | Yes | Yes | 9 | High |
| Schmid et al., 2022 | Yes | Yes | No | Yes | No | No | No | Yes | NA | Unclear | Yes | 5 | Moderate |
| Werner et al., 2022 | Yes | Yes | Yes | No | No | No | Unclear | Yes | NA | Yes | Yes | 6 | Moderate |
| Ruby et al., 2015 | Yes | Yes | No | Yes | Yes | Unclear | Yes | Yes | NA | Yes | Yes | 8 | High |
| Lin et al., 2022 | Yes | Yes | Yes | No | No | No | No | Yes | NA | Yes | Yes | 6 | Moderate |
| Dobiesz et al., 2022 | Yes | No | Unclear | No | No | No | Yes | Yes | NA | Yes | Yes | 5 | Moderate |
| Beek et al., 2017 | Yes | Yes | Unclear | Yes | Yes | Unclear | Unclear | Yes | NA | Yes | Yes | 7 | Moderate |
| Abujaber et al., 2022 | Yes | Yes | Yes | Yes | Yes | Unclear | Yes | Yes | NA | Yes | Yes | 9 | High |
| Bowsher et al., 2021 | Yes | Yes | No | Yes | No | No | Unclear | Yes | NA | Unclear | Yes | 5 | Moderate |
| Winders et al., 2021 | Yes | Yes | Yes | Yes | Yes | Unclear | Yes | Yes | NA | Unclear | Yes | 8 | High |

**Key: a** yes answer out of 10

Low (0-4); Moderate (5-7); High (8-10)
