# Supplementary material for: Health system strengthening in fragile and conflict-affected states: A review of systematic reviews
Source: PLoS One. 2024 Jun 14;19(6):e0305234. doi: 10.1371/journal.pone.0305234 (PMC11178226; doi:10.1371/journal.pone.0305234)
Supplement: Attachment — Submitted filename: Response to Reviewers.docx [file pone.0305234.s005.docx]

15 February 2024

The Editor

PLOS ONE

Dear Sir/Madam,

**Health system strengthening in fragile and conflict-affected states: a review of systematic reviews.**

Birke Bogale, Sasha Scambler, Aina Najwa Mohd Khairuddin, Jennifer E. Gallagher

On behalf of my co-authors, I would like to thank you and the reviewers for most helpful comments on the above paper. We have reviewed the reviewer’s suggestions and taken on board their comments while updating our manuscript. As per your suggestion, we have made the required changes in the results, discussion, and limitations sections. We have elaborated the bibliometrics section and quality appraisal in the main text of the manuscript and added a table in an online annex detailing the quality appraisal. We have also updated our eligibility criteria and explained why Jaung et al was not eligible in the response to reviewers, below. There are a range of definitions and indices available in relation to fragile and conflict affected states, and different systematic reviews have adopted heterogeneous definitions. To address this, we have included systematic reviews conducted in the various contexts of fragility and conflict by assessing them case by case against the available literature. Moreover, we have conducted a thorough proofreading for minor errors and grammatical mistakes and rewritten the discussion.

Regarding our funding information, we would like to update it as **‘This research is part of BB’s PhD study funded by King’s College London via ‘Africa International PGR Scholarships 2021-22’ (URL: https://www.kcl.ac.uk/study-legacy/funding/africa-international-pgr-scholarships). However, there is no funding specific to the present research paper**’.

Please note that, we have exchanged the order of our figures in the manuscript and uploaded the documents with their updated label.

We feel that the additional work has enhanced the quality of this paper. Our response to each of the points raised is outlined below.

We look forward to hearing from you.

Yours faithfully,

Birke Bogale (Lead author)

On behalf of the coauthors

**Reviewers' comments and responses**

**Reviewer #1:** This review of systematic reviews sought to explore health system strengthening in fragile and conflict-affected states and synthesize the evidence from published literature. It addresses an important topic. The design is appropriate, and it is well conducted and nicely written. The conceptual framework is appropriate and helpful.

***Author’s Response (AR):*** *Thank you.*

Results:

I find some of the results a bit odd. For example, the Ruby review being included when none of the papers in Ruby’s review seemed specifically about health systems? Related to this, why was a review on models of NCD care in humanitarian crises by Jaung et al ( see doi: 10.1093/heapol/czab007 ) not eligible when it seems much more related to health systems than Ruby et al.? I can also think of many systematic reviews on other health outcomes (e.g. mental health interventions among conflict-affected populations) that would appear as relevant as Ruby to health systems but were not considered eligible for this review. For example, https://gh.bmj.com/content/4/5/e001484.info and https://doi.org/10.1186/s13033-020-00431-1. I appreciate there’s a conceptual fuzziness with health systems that can make it difficult to determine inclusion/exclusion criteria of individual papers but I think this needs better explanation. It may also be worth noting this fuzziness as a potential limitation?

***AR:*** *We aimed to look at health system strengthening in its broad form, and the utilised framework contributed to our eligibility criteria. We excluded papers on specific health interventions or those solely targeting provisions of care for individual health conditions rather than more generalised/wider approaches. We also excluded those targeting specific population groups, such as age groups, and professions.*

*We agree that the studies included in Jaung et al., (2021) are related to health system and its inclusion may have added value to the findings of our review. However, the subject of this paper, ‘models of care specific to patients with diabetes and hypertension’ was regarded as too condition specific. Our inclusion/exclusion criteria excluded these condition specific papers to enable us to look at papers that looked at health system strengthening more broadly.*

There was little information on how the findings from the eligible reviews relate to different phases of crisis (i.e. acute, chronic, prolonged, post-conflict/crisis rebuilding). These temporal aspects related to health systems are really important and more investigation would have been helpful. Could the findings in relation to different phases be brought out more?

***AR:*** *The evidence on the temporal differences of the findings in the included systematic reviews is very limited. The systematic reviews mostly did not consider the temporality, and some papers acknowledged this as a limitation. However, we tried to integrate this aspect in the review where the information was available. We have also provided more explanations in the response to reviewer 2.*

No results on quality appraisal are given apart from in Table 3. They should be given as summarized form in the main text of the manuscript and then in more detail using a Table in an online annex.

***AR:*** *We have included a summary in the results section, also added more detail. An online annex (S4 Annex) of the detail has also been provided.*

Discussion:

The Discussion section is generally quite nicely written. However, it is quite repetitive of the Results section (including re-using a lot of the citations from the Results section). It would be more helpful if the Discussion section could put the findings in a broader context – for example, how it relates humanitarian guidelines and policies, findings of reviews of health systems in stable LMIC settings.

***AR:*** *Thank you, this is really helpful, we have rewritten the discussion section.*

Limitations section:

I found most of the Limitations section quite impenetrable.

For example, in the second limitation (which is a valid and important one), the first sentence doesn’t seem to make any sense (“the systematic reviews included literature from countries with a history of being classified as FCAS or not, confirming the possibility of fragility manifesting within any setting or system”). What does this actually mean? Perhaps it could be more clearly written.

For example, I didn’t understand the third limitation (“this review may have only included HSS initiatives focused on the earlier stages of crises and may lack coverage of all aspects or the later stages of HSS of FCAS”). Shouldn’t the authors be able to determine this by looking at the papers, rather than just speculate on it?

For example, I also didn’t understand this. “Fragile states may also mean LMICs because of the complexity of the fragile situations which slows down their progress”. How is this a limitation? All fragile states are LMICs aren’t they? It’s also poorly written.

I also didn’t get why “the Lancet’s high-quality health system framework may have indirectly targeted FCAS”. Why would it have done this and why is this a limitation?

***AR:*** *Thank you. This is very helpful; the section has now been rewritten to make it clearer.*

**Reviewer #2:** This is a very interesting and comprehensive review of reviews, however, the manuscript could be improved substantially.

Minor comments

Abstract: you note the importance of HSS and quality- but do not define quality of what - though I can see that later you reference the high-quality HS framework.

***AR:*** *Thank you, we have specified it as ‘quality in health systems’ for more clarity.*

Line 62 introduction: humanitarian crises are not just disasters - remove the bracket

***AR:*** *The term ‘disaster’ in the specified section intended to show that the terms humanitarian crisis and humanitarian disasters are used in the literature interchangeably. However, we have removed the bracket as per your suggestion.*

Line 96 - Assume you mean Figure 1

***AR:*** *Thank you for pointing this out, it is now corrected as per the order of appearance. We have also resubmitted the diagram that appears in the manuscript first renamed as ‘Fig 1’.*

Line 109 - PRISMA is a reporting guideline, not a ‘how to do a systematic review’ guideline, please correct

***AR:*** *Thank you. It is corrected.*

Line 233 - do you mean lack of accountability?

***AR:*** *‘Perceived lack of accountability’, explains it better. It is changed, thank you.*

Line 250 - do you mean decentralization supported health system reform? This needs to be unpacked a bit, not clear

***AR:*** *Yes, it is changed to ‘decentralisation supported health system reform’.*

Lines 618-629 - If I understand correctly, you wish to say: findings of included reviews predominantly focus on inputs but neglect to focus on processes/outcomes etc. The first 2 lines are confusing and can be cut in the paragraph, and the rest really needs to be reworked - and ideally contextualized: do the included systematic reviews present their data according to WHO building blocks? What framings do they use?

***AR:*** *Thank you. The discussion section is rewritten. We did not include the framings the systematic reviews have used since they studied different areas of health system and follow different approaches; also, not all considered using available health system frameworks.*

Lines 635 - what framework?

***AR:*** *It is specified as ‘the high-quality health system framework’.*

Lines 638-640 seem like a big over generalization. My review stops here.

***AR:*** *It was based on existing literature, which may not be explicitly written. We have removed it in the current draft.*

Major comments

A more thorough bibliometrics section should be included at front - there is too little here - how many of the countries included fall into what type of FCAS definition? What are the broader problems that these studies consider? What is the nature of the fragility discussed etc.

***AR:*** *We have added more details in the bibliometric section, including the details of the countries in terms of the world bank’s list of fragile and conflict-affected situations. However, we only manually extracted the countries mentioned in the systematic reviews. Our list of countries may not represent all the studied countries, as the systematic reviews also used collective names/terms and different ways of reporting.*

For each of your main sections please clearly list how many reviews considered that topic

***AR:*** *We have included the number of reviews in each section.*

Governance section: more broadly, this section and others would benefit from a figure or diagram where you clearly identify main challenges and HSS approaches that have worked. The text here is quite descriptive and dry, which is understandable, but at the minute it is very difficult to follow.

***AR:*** *Thank you for the comment, we believe the key findings/ HSS initiatives column in ‘Table 3’ and ‘S2 Annex’ containing summaries of the main findings helps in following the results.*

*We have updated the results section and the summary of the ‘governance’ section to make it easier to follow. We have also included an additional table (Table 4) with the specified challenges, and interventions that were reported highlighted.*

My review stopped mid-way through discussion as there are quite sweeping statements made here that do not always link back clearly to your findings. Across your presentation of findings there is a tendency to treat all FCAS as a uniform group - this is actually quite damaging. I.e. active conflict is very different to past conflict, which in turn is very different to a localized natural disaster or to persistent climatic change (e.g. repeated drought). Was there any analysis done to unpack any potential differences in findings according to the diverse nature of the FCAS in question? The health workforce section is the only one that teases out these differences, so maybe try that for the other sections too. How do the reviews you included deal with this heterogeneity with the FCAS? What definitions did they employ for inclusion/exclusion?

***AR:*** *In this paper we attempted to map out the findings in terms of the stages and contexts of the situation. The systematic reviews included in this paper vary in the types of fragility and the elements of health system strengthening addressed, as well as the diverse criteria used in the included systematic reviews. For example, some papers utilised ‘humanitarian setting/crisis’ to describe collective contexts or settings such as conflict/post-conflict, natural disasters, and displaced population/refugees, while others described them in terms of FCAS and synthesised their data accordingly. In addition, while some papers presented the findings in terms of specific contexts such as conflict-affected, post-conflict or displaced populations, others were described collectively as humanitarian or fragile contexts. The temporality of the interventions is also not addressed in most reviews. However, we have included all reviews studying contexts related to FCAS that are specified in the paper regardless of their eligibility criteria, and the definitions or metrics used. Narrowing down the inclusion criteria may have led to losing relevant information/evidence.*

*Most of the evidence related to the workforce came from ‘conflict-affected’ contexts (during or post-conflict) and involves relatively homogenous or interconnected subject areas unlike all the other topics. Overall, our presentation of the findings has been shaped by how the included systematic reviews treated and categorised the countries/contexts included in their papers and how they presented their findings.*
